# Estimation of cervicocephalic kinesthetic perception and its correlation with fall risk in adults with diabetes and without diabetes experiencing cervical pain: A comparative study

**DOI:** 10.1371/journal.pone.0323457

**Published:** 2025-06-12

**Authors:** Shilpi Anand, Divya Aggarwal, Sahar Zaidi, Himani Kaushik, Irshad Ahmad, Debjani Mukherjee, Ghada Mohammed Koura, Emadeldin Mohammed Mukhtar, Nasrin Mansuri, Fuzail Ahmad, Ravi Shankar Reddy, Muhammad Sufyan

**Affiliations:** 1 School of Medical & Allied Sciences, GD Goenka University, Haryana, India; 2 Department of Physiotherapy, Manav Rachna International Institute of Research and Studies, Faridabad, India; 3 Department of Physiotherapy, Jamia Hamdard, New Delhi, India; 4 Banarsidas Chandiwala Institute of Physiotherapy, Guru Gobind Singh Indraprastha University, Delhi, India; 5 Central Labs, King Khalid University, AlQurra’a, Abha, Saudi Arabia; 6 Program of Physical Therapy, Department of Medical Rehabilitation Sciences, College of Applied Medical Sciences, King Khalid University, Abha, Saudi Arabia; 7 Department of Radiological Sciences, College of Applied Medical Sciences, King Khalid University, Abha, Saudi Arabia; 8 Department of Clinical Laboratory Sciences, College of Applied Medical Sciences, King Khalid University, Abha, Saudi Arabia; 9 Rehabilitation Sciences Department, College of Applied Sciences College, Almareefa University, Dirirya, Riyadh, Saudi Arabia; 10 Hamdard Institute of Medical Sciences and Research, Hamdard University, New Delhi, India; University of Limpopo, SOUTH AFRICA

## Abstract

**Aims:**

This study aimed to evaluate the relationship between cervicocephalic kinesthesia sensation and the incidence of falls in adults with Type 2 Diabetes Mellitus (DM-2) experiencing cervical pain.

**Methods:**

The research was conducted between January 1, 2022, and August 15, 2022. A total of 60 participants were included, with an average age of 26.03 ± 3.45 years and an average BMI of 21.4 ± 2.05 kg/m². Participants were divided into two groups: an experimental group of adults with DM-2 and cervical pain, and a control group without DM-2 but with cervical pain.

**Results:**

Head repositioning errors were significantly higher in the experimental group for right-side and left-side lateral flexion (P = 0.002). Forward flexion also showed a significant difference between groups (P = 0.006). However, no significant differences were observed between the groups in extension (P = 0.589). No considerable differences were noted in the Berg Balance Scale (BBS) and Neck Disability Index (NDI) scores between the groups.

**Conclusions:**

Individuals with DM-2 experiencing neck pain exhibited minimal errors in head repositioning tests, yet no substantial alterations were evident in NDI and BBS scores.

## 1. Introduction

Type 2 Diabetes Mellitus (DM-2), a prevalent metabolic disorder, affects approximately 422 million individuals globally [1]. The disease manifests as heightened blood sugar levels, stemming from insufficient insulin production or cellular resistance to existing insulin [1]. Projections indicate a rising trend in diabetes prevalence from 2.8% in 2000 to an anticipated 4.4% by 2030, signifying a looming global health crisis. Obesity, sedentary lifestyles, and inadequate physical activity are primary contributors to this escalating diabetes burden [1].

The impact of diabetes goes beyond its prevalence. It ranks as the 18th highest common non-lethal ailment worldwide, but the potential for premature mortality threatens to elevate its standing to the 7th position between 2016 and 2040 issue [2]. This trajectory prompts considerations for global target scenarios, acknowledging the escalating economic ramifications of this health issue [2]. Despite its decline in mortality ranking, diabetes retains its position as the ninth-most disabling ailment over time, as per data derived from a Spain-based study between 1990 and 2016. Such trends signify not just a health concern but also an economic and societal challenge [2].

Furthermore, diabetes poses a significant risk to patients’ well-being, impacting their quality of life and causing chronic musculoskeletal pain. Studies suggest a notable prevalence of pain and a higher body mass index (BMI) correlating with diminished quality of life, reduced physical function, and limited engagement in physical activities among diabetic patients [3].

The complications stemming from diabetes significantly burden healthcare systems, with chronic somatic pain, particularly in the musculoskeletal system, being one of the major concerns. Conditions like low back pain and neck pain represent primary complications linked to diabetes. Global Burden Disease data from 2017 revealed that low back pain topped the list of health burdens, with diabetes ranking fourth, and neck pain ninth for females and eleventh for males [2]. This escalating pain prevalence, up from 1.7 to 2.1 times over time and when compared to the general population, underscores the pressing need for effective management strategies [2].

The cervical spine is intricately connected to various regions of the body, influencing not only local but also distant structures through its extensive neural, muscular, and vascular connections. Dysfunction in the cervical spine can have widespread implications, such as contributing to tension headaches, where altered cervical muscle tone and joint dysfunction refer pain to the craniofacial region [4]. Additionally, cervical spine changes can impact masticatory muscle function, leading to impaired jaw movements and temporomandibular joint disorders [5]. These connections underscore the importance of evaluating cervical spine health in patients presenting with symptoms beyond the immediate neck area. Additionally, chronic neck pain associated with cervical spine issues remains a considerable concern. Studies investigating cervicocephalic kinesthesia sensitivity among patients with chronic neck pain suggest functional alterations in muscle spindle receptors, possibly attributed to muscle and articular dysfunction. This altered sensitivity might contribute to persistent neck pain, especially following cervical injuries like whiplash [6,7].

Sensory deficits, particularly in the somatosensory domain, significantly impact postural stability in individuals with diabetes. These impairments in somatosensation, known to alter afferent fiber characteristics, are correlated with increased postural instability in individuals with diabetic neuropathy [8].

The relationship between sensory deficits and compromised balance control is a critical area of study. Factors such as diminished vision, muscle weakness, vestibular disorders, and somatosensory deficits contribute to balance impairments and people with diabetes are more likely to experience falls [9].

Understanding the relationship between sensory perceptions and fall incidences in diabetes patients suffering from neck pain is crucial. This gap in research emphasizes the need for interventions focusing on kinaesthetic sensation, cervical receptors, and fall prevention strategies in this vulnerable population. Therefore, the study aimed to evaluate the relationship between cervico-cephalic kinesthesia sensation and the incidence of falls in diabetes mellitus adults having cervical pain. We hypothesize that individuals with DM-2 experiencing cervical pain will exhibit significant impairments in cervicocephalic kinesthesia and an increased risk of falls compared to individuals without DM-2, due to the compounded effects of diabetic neuropathy and cervical dysfunction.

## 2. Methodology

The research investigation adopted a cross-sectional design and took place from January 1, 2022 to August 15, 2022 at the Department of School of Medical & Allied Sciences, with approval from the Institutional Ethics Committee (I.E.C.) (Proposal Number: ECM#2021−6010). The research was conducted in accordance with the Helsinki Declaration (2013). Prospective participants underwent rigorous screening to ascertain their eligibility for study inclusion. Participants in the experimental group were diagnosed with DM-2 within the past six months, ensuring the study focused on the early effects of diabetes on kinesthetic perception and fall risk. Sixty individuals, consisting of both genders, met the inclusion criteria, age group of 18–35 years, a normal body mass index (BMI) ranging between 18 and 24 kg/m², persistent asymptomatic cervical pain lasting for at least three months, and a confirmed diagnosis of DM-2 (diagnosed within the past six months). Before participation, everyone who was enrolled asked to give their written approval. The age range of 18−35 years was selected because of the increasing incidence of DM-2 in younger adults, driven by factors such as rising obesity rates and sedentary lifestyles. The age range of 18–35 years was selected due to the increasing prevalence of DM-2 in younger adults, primarily attributed to rising obesity rates and sedentary lifestyles [10]. Previous studies highlight that early-onset DM-2 exhibits a more aggressive disease progression, leading to higher risks of sensorimotor impairments, including deficits in kinesthetic perception and postural instability, compared to later-onset cases [11]. This age group represents a growing demographic in DM-2, with distinct clinical challenges and disease trajectories. Studying this cohort allows for the assessment of early impacts of DM-2 on cervicocephalic kinesthesia and fall risk, providing insights that may differ from older populations typically associated with DM-2. Exclusion criteria consisted of subjects with cervical radiculopathy, cervical traumas, tumours, a history of the upper limb, hand, or cervical surgeries, carpal tunnel syndrome, rheumatoid arthritis, vestibular issues, neuropathies, hand deformities, athletes, a history of dizziness or vertigo, and any other musculoskeletal, neurological, or endocrine concerns.

Two groups of participants were formed: a) Adults with Diabetes Experiencing Cervical Pain served as an experimental group and; b) Adults without Diabetes Experiencing Cervical Pain served as a control group. The participants were asked to give their written consent for publishing the data and pictures.

### 2.1. Study procedure

**Fig 1:**
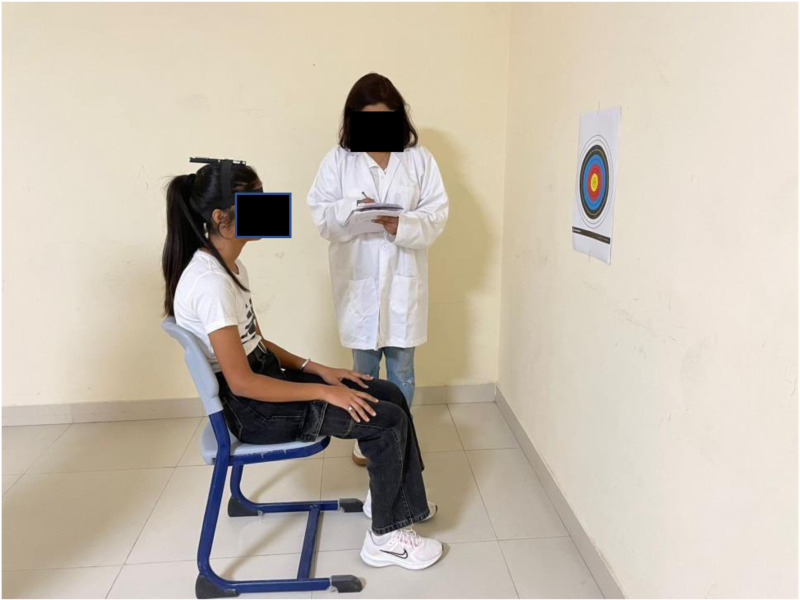
Subject undergoing assessment using the Head Repositioning Error Test (HRT).

Head Repositioning Test (HRT): The HRT represents a cost-effective and straightforward approach for quantifying head-to-trunk repositioning across various head orientations and anatomical planes (e.g., transverse, sagittal). The reliability of the head repositioning test is notably high (ICC, 0.81) with concurrent validity [12]. Detailed instructions on the HRA procedure were provided to each one of the participants. The subjects wore sleeping masks that blinded them and instructed them to maintain closed eyes throughout the test. Participants were positioned in a seated posture with back support, making sure the knees and hips form a right angle and that the feet are flat on the floor. The target paper for the test was affixed to the wall approximately 90 cm from the subject’s seat and adjusted according to the individual’s height. A laser pointer was fixed at the highest point of the subject’s head. Initially, with eyes open to familiarize themselves with the test, participants were asked to center their heads on the target paper. Subsequently, with closed eyes, subjects were directed to move their head in four directions—right, left, upward, and downward—as far as possible, endeavoring to return to the starting point. The subject’s movements in each direction were marked using colored stickers, and 10 consecutive repetitions were made for each directional movement. The therapist then measured the distance between the initial center point & the farthest point reached by the subject using a ruler. Normal relocation was defined as within 7 cm or less than 4.5 degrees (horizontal) from the starting point, while any deviation beyond this range was considered an abnormal error (Fig 1).

**Fig 2:**
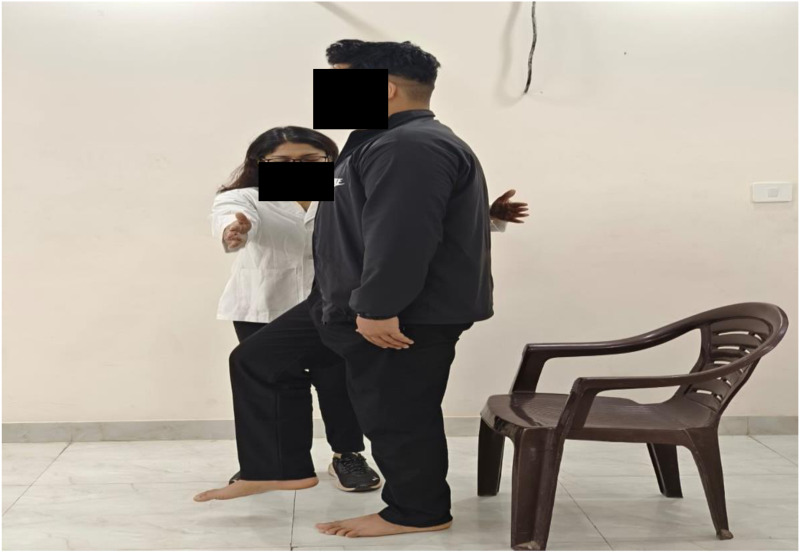
Evaluation of Balance Using the Berg Balance Scale.

Risk of Fall Measurement: The Berg Balance Scale (BSS) assessment serves as the gold standard for evaluating both static and dynamic balance abilities. Widely employed in clinical and academic settings, this tool quantifies therapeutic efficacy and provides a quantitative depiction of functional status. Comprising a 14-item scale for basic balancing activities—transfers, simple object retrieval movements, posture changes, and various postures—the performance of each test is graded on a scale from 0 (unable) to 4 (independent). Lower scores indicate more severe balance impairments [13]. The Berg Balance Scale was utilized to assess the balance test (Fig 2).

**Fig 3:**
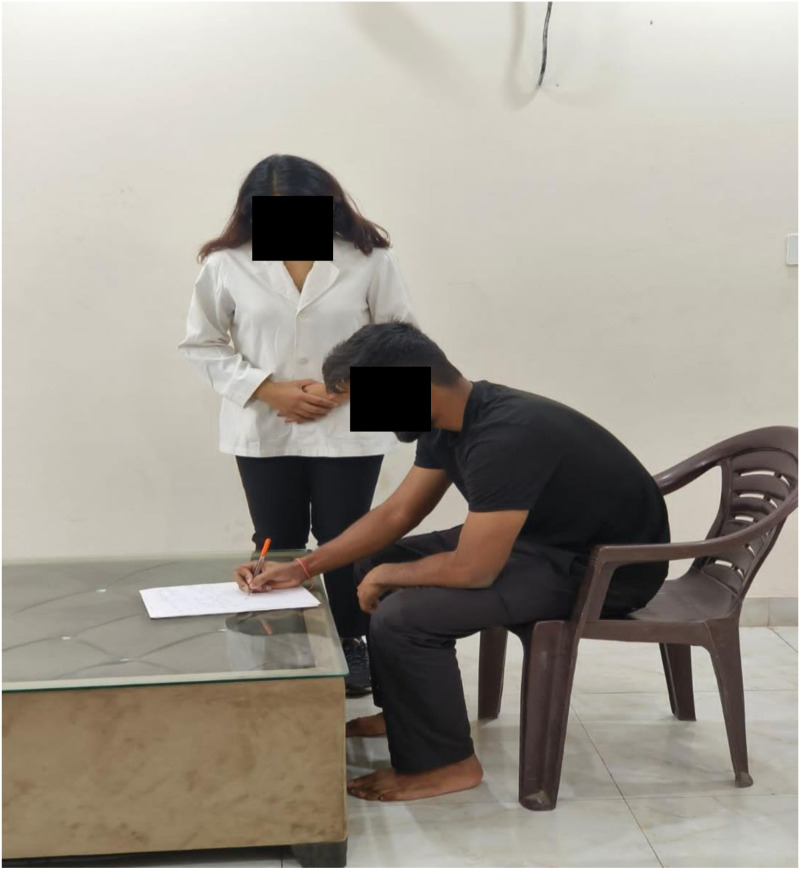
Administration of the Neck Disability Index Questionnaire.

Self-Rated Disability Measurement due to Neck Pain: The Neck Disability Index (NDI), introduced in 1991, stands as the most extensively employed tool for self-rated disability assessment in individuals experiencing neck pain [14,15]. This self-report questionnaire comprises 10 items, covering aspects such as pain intensity, personal care, lifting, work, headaches, concentration, sleeping, driving, reading, and recreation. Responses for each item range from 0 (no disability) to 5 (complete disability), with the total score varying from 0 to 50 [16]. The stability of the neck was assessed using the Neck Disability Index (Fig 3).

The sample size calculation was based on effect size data from the study by Lima Florencio [2], which reported significant differences in postural stability measures between diabetic and non-diabetic groups. An effect size of 1.08 was used, reflecting the expected substantial differences in head repositioning errors due to the pronounced impact of diabetic neuropathy on sensorimotor function. The alpha level was set at 0.05 to minimize the risk of Type I error, while a power of 0.95 was chosen to ensure a high probability of detecting true effects, given the critical nature of our study outcomes related to fall risk. The calculations were performed using G Power 3.1.9.4 for a one-tailed t-test, which was appropriate for our directional hypothesis expecting greater impairment in the experimental group compared to the control group.

Data analysis was executed using SPSS 21.0 software and managed within an Excel spreadsheet. For the descriptive analysis, a 95% confidence interval was used, and a one-tailed t-test was considered statistically significant if the p-value was less than 0.05 (5%).

## 3. Results

The control group (n = 30) and experimental group (n = 30) were compared across several variables (Table 1). Significant differences were observed in age (control: 23.8 ± 1.32 years vs. experimental: 28.26 ± 4.33 years, p = 0.003), height (control: 5.51 ± 0.27 ft vs. experimental: 5.64 ± 0.35 ft, p = 0.045), weight (control: 61.3 ± 9.86 kg vs. experimental: 69.03 ± 8.76 kg, p = 0.012), and BMI (control: 21.4 ± 2.3 kg/m² vs. experimental: 23.6 ± 2.8 kg/m², p = 0.018). There was no significant difference in sex ratio between the groups (control: 16 females, 14 males; experimental: 11 females, 19 males; p = 0.21).

**Table 1:** Characteristics of Control and Experimental Group.

Variable	Control Group (n = 30)	Experimental Group (n = 30)	p-value
Sex ratio (F:M)	16:14	11:19	0.21
Age (years)	23.8 ± 1.32	28.26 ± 4.33	0.003
Height (ft)	5.51 ± 0.27	5.64 ± 0.35	0.045
Weight (kg)	61.3 ± 9.86	69.03 ± 8.76	0.012
BMI (kg/m²)	21.4 ± 2.3	23.6 ± 2.8	0.018

F: Female, M: Male, BMI: Body Mass Index, ft: feet, kg: kilograms.

The descriptive analysis of the control and experimental groups shows significant differences across multiple variables (Table 2). For the Head Repositioning Test (HRT), both right and left side flexion, as well as flexion and extension, demonstrated large effect sizes, with Cohen’s d values ranging from 2.27 to 3.60, indicating substantial improvements in the experimental group compared to the control. The t-values for these variables ranged from 7.32 to 10.25, all reaching statistical significance with p-values ≤ 0.002. Additionally, the BBS and the Neck Disability Index (NDI) also showed statistically significant differences between groups, with moderate effect sizes (Cohen’s d of 1.36 and 1.47, respectively) and p-values ≤ 0.004. These findings suggest that the experimental intervention resulted in significant improvements in both motor and balance outcomes.

**Table 2:** Descriptive Analysis of Variables of Control and Experimental Group.

Variable	Control Group Mean ± SD	Experimental Group Mean ± SD	Effect Size (Cohen’s d)	95% CI for Mean Difference	t-value	p-value
HRT: RSF	5.49 ± 1.06	8.14 ± 0.42	2.93	2.35, 3.0]	8.45	0.001
HRT: LSF	4.87 ± 1.38	7.68 ± 0.43	2.27	2.01, 2.92	7.32	0.002
HRT: FLEXION	4.77 ± 0.85	7.29 ± 0.70	3.19	2.41, 3.56	9.12	0.001
HRT: EXTENSION	4.15 ± 0.90	7.08 ± 0.50	3.60	2.61, 3.77	10.25	0.001
BBS	55.36 ± 1.18	50.53 ± 4.52	1.47	3.12, 6.11	5.64	0.003
NDI	46.96 ± 1.84	43.86 ± 2.51	1.36	1.88, 4.01	4.79	0.004

Note: BBS: Berg Balance Scale, HRT: Head Repositioning Test, LSF: Left Side Flexion, NDI: Neck Disability Index, RSF: Right side Flexion.

## 4. Discussion

This study aimed to evaluate the relationship between cervicocephalic kinesthetic perception and fall risk in individuals with DM-2 experiencing cervical pain. The results revealed significant differences in head repositioning errors between the diabetic and non-diabetic groups, particularly in right-side flexion, left-side flexion, and forward flexion, suggesting impaired proprioceptive control in individuals with DM-2. However, no significant differences were observed in NDI or balance performance (BBS) scores between the groups, indicating that while cervicocephalic proprioceptive deficits exist in diabetic individuals, their functional impact on overall balance and disability may be influenced by additional factors such as peripheral neuropathy, vestibular function, and postural compensations. These findings align with existing literature highlighting proprioceptive impairments in diabetes but underscore the need for a more comprehensive assessment of multisensory contributions to postural control and fall risk in this population.

Although the gender distribution was not statistically different between the control and experimental groups (p = 0.21), a significant difference in mean age was observed (p = 0.003), with the experimental group being older. Since proprioception and balance control can be influenced by age, even within the 18–35 range, this age difference may have contributed to the higher head repositioning errors and fall risk scores observed in the diabetic group. Future studies should consider age-matching or applying statistical controls to account for age as a confounding variable.

In recent researchers have compared healthy individuals with those who had type 2 diabetes (T2D) to see how well they were able to sense the precise position of their cervical joints. They also looked for a correlation between glycated hemoglobin levels and this sensation in the T2D population. Their study concluded that individuals with T2D exhibited considerable impairment in cervical joint position sense. Moreover, they observed a significant negative correlation between cervical joint position sense and HbA1c levels [17]. The study found that compared to the healthy group, people with type 2 diabetes had a lot more errors when repositioning their cervical joints in several directions, including as flexion, extension, left rotation, and right rotation [17]. Furthermore, in people with type 2 diabetes, there was a moderately favorable connection between HbA1c and the ability to sense the position of the cervical joints across all assessed directions [17]. These findings resonate with earlier research but warrant further justification.

Diabetic neuropathy causes neurological effects such a lack of oxygen, small-fiber degeneration, and decreased sensations of pain feedback, which may explain why people with T2D have a diminished feeling of cervical joint position. Previous studies have also indicated worse joint position sense in individuals with T2D, including studies examining knee and hip joint proprioception [18–21]. Our investigation aligns with these prior findings. However, further justifications are necessary.

An investigation of the role of chronic pain as a mediator between functional balance and cervical proprioception in older adults suffering from chronic neck pain was carried out in 2023 [22]. They reported that people in their later years who suffer from persistent neck discomfort exhibited impaired cervical proprioception and functional balance compared to asymptomatic individuals. Notably, these individuals demonstrated increased cervical joint position errors in all directions assessed, indicating poor cervical proprioception [23–25]. Additionally, they experienced significantly diminished functional balance in comparison to asymptomatic individuals. The study highlighted a strong correlation between BBS test scores, Timed Up and Go (TUG) scores, and cervical joint position errors in chronic neck pain patients [22].

In a recent pilot study, individuals suffering from nontraumatic persistent pain in the neck were evaluated for postural balance and cervicocephalic kinesthetic sensitivity. Their findings indicated increased global repositioning errors in the experimental group, particularly in flexion, compared to the control group. However, no general impairment in postural balance was observed across the groups [26].

The inclusion of Body Mass Index (BMI) comparisons between the control and experimental groups highlights its relevance as a contributing factor to the observed differences in cervicocephalic kinesthesia and fall risk. The large effect sizes calculated for BMI, head repositioning errors, and other key variables indicate both statistical significance and practical importance, emphasizing the potential impact of diabetic neuropathy on sensorimotor function and postural stability. These findings underscore the need for targeted interventions in individuals with DM-2, particularly those with higher BMI, to mitigate the associated risks of impaired kinesthesia and increased fall likelihood.

Fall risk was assessed using the Berg Balance Scale (BBS), a validated tool for evaluating balance impairments; however, no separate test was conducted to determine whether falls were primarily due to cervical muscle dysfunction or postural changes. While cervicocephalic kinesthetic deficits can contribute to postural instability, diabetic peripheral neuropathy, which affects sensory feedback, remains a key factor. The lack of direct assessments for peripheral neuropathy, such as nerve conduction studies or monofilament testing, is a limitation, as peripheral sensation was not explicitly evaluated. Given that diabetic neuropathy significantly impacts sensory feedback and balance, future research should incorporate standardized peripheral sensory evaluations to better differentiate the effects of neuropathy from cervicocephalic proprioceptive impairments in DM-2 individuals.

In younger individuals with DM-2, sensory impairments due to diabetic neuropathy are often mild and may be reversible with improved glycemic control, as suggested by Gibbons and Freeman, [27]. Since our study included participants diagnosed with DM-2 within the past six months, it is possible that neuropathy was in its early stages and did not significantly contribute to balance impairments. While this study primarily focused on cervicocephalic kinesthesia and fall risk, future research should investigate the progression and reversibility of sensory deficits in younger diabetic populations, considering the impact of metabolic control on neuropathy outcomes.

Additionally, vertigo was not explicitly assessed; however, participants with a history of dizziness or vertigo were excluded to minimize confounding variables. While cervicocephalic kinesthetic deficits may impact postural stability, falls in DM-2 individuals are often multifactorial, involving peripheral neuropathy, vestibular dysfunction, and musculoskeletal impairments. The absence of vestibular function assessments is a limitation, and future studies should integrate comprehensive vestibular evaluations to better delineate the role of cervical proprioceptive dysfunction in fall risk.

### 4.1. Limitations & future suggestions

Our study similarly identified significant differences in head repositioning error between groups, yet no correlations were found with NDI and BBS. Nevertheless, several limitations were encountered, including small sample size, single-time observational design, lack of randomization, and the oversight of blood sugar levels in the inclusion criteria. Further investigations are recommendations to corroborate these findings effectively. This study did not include a separate postural analysis to objectively assess postural deviations. While cervical proprioceptive deficits can influence postural control, the findings were based on the Head Repositioning Test and BBS without direct postural assessment. The absence of postural analysis is a limitation, and future research should incorporate objective postural assessment methods, such as digital posturography, to better understand the relationship between postural changes, proprioceptive deficits, and fall risk in individuals with DM-2.

## 5. Conclusion

Individuals with DM-2 experiencing cervical pain demonstrated greater errors in cervicocephalic kinesthetic perception compared to non-diabetic individuals, yet no significant differences were found in Neck Disability Index (NDI) or BBS scores. The study highlights the need for comprehensive assessments, including peripheral sensation, vestibular function, and postural analysis, to fully understand the multifactorial contributors to fall risk in DM-2. Clinically, early identification of proprioceptive impairments can facilitate the implementation of targeted rehabilitation strategies, such as proprioceptive training, postural corrections, and fall prevention programs, to reduce fall risk and enhance stability in diabetic individuals with cervical dysfunction. Further research with larger sample sizes and longitudinal follow-ups is needed to validate these findings and develop effective intervention strategies.

## Supporting information

S1 File Raw_Data_Cervicocephalic_Study. (XLSX)
