# Correction: Estimation of cervicocephalic kinesthetic perception and its correlation with fall risk in adults with diabetes and without diabetes experiencing cervical pain: A comparative study

**DOI:** 10.1371/journal.pone.0344597

**Published:** 2026-03-09

**Authors:** Shilpi Anand, Divya Aggarwal, Sahar Zaidi, Himani Kaushik, Irshad Ahmad, Debjani Mukherjee, Ghada Mohammed Koura, Emadeldin Mohammed Mukhtar, Nasrin Mansuri, Fuzail Ahmad, Ravi Shankar Reddy, Muhammad Sufyan

There is an error in affiliation 9 for author Fuzail Ahmad. The correct affiliation 9 is: Respiratory Care Department, College of Applied Sciences, Almareefa University, Dirirya, Riyadh, Saudi Arabia.

The following information is missing from the Acknowledgement statement: Fuzail Ahmad would like to thank AlMaarefa University, Diriya, Riyadh, Saudi Arabia for supporting this research.
